# Performance evaluation of 3 serodiagnostic peptide epitopes and the derived multi-epitope peptide OvNMP-48 for detection of *Onchocerca volvulus* infection

**DOI:** 10.1007/s00436-019-06345-3

**Published:** 2019-05-14

**Authors:** Ole Lagatie, Ann Verheyen, Erik Nijs, Linda Batsa Debrah, Yaw A. Debrah, Lieven J. Stuyver

**Affiliations:** 1 Global Public Health, Janssen R&D, Turnhoutseweg 30, 2340 Beerse, Belgium; 2 Kumasi Centre for Collaborative Research into Tropical medicine, Kwame Nkrumah University of Science and Technology, Kumasi, Ghana; 3 Faculty of Allied Health Sciences, Kwame Nkrumah University of Science and Technology, Kumasi, Ghana

**Keywords:** *Onchocerca volvulus*, River blindness, Onchocerciasis, Serology, Linear epitope

## Abstract

Current diagnostic tools to determine infection with the helminth parasite *Onchocerca volvulus* have limited performance characteristics. In previous studies, a proteome-wide screen was conducted to identify linear epitopes in this parasite’s proteome, resulting in the discovery of 1110 antigenic peptide fragments. Here, we investigated three of these peptides using peptide ELISA’s and evaluated their sensitivity and specificity. Epitope mapping was performed, and peptides were constructed that contained only the minimal epitope, flanked by a linker. Investigation of the performance of these minimal epitope peptides demonstrated that all three of them have a specificity (as defined by lack of response in non-helminth-infected individuals) of 100%, low cross-reactivity (5.6%, 5.6%, and 9.3%, respectively), but low sensitivity (36.9%, 46.5%, and 41.2%, respectively). Some cross-reactivity was observed in samples from individuals infected with soil-transmitted helminths or *Brugia malayi*. Combining these three minimal epitopes in a single peptide, called OvNMP-48, resulted in a performance that exceeded the sum of the individual epitopes, with a sensitivity of 76.0%, a specificity of 97.4%, and a cross-reactivity of 11.1%. Cross-reactivity was observed in some STH and *Brugia malayi*-infected individuals. This work opens the opportunity to start exploring how these novel linear epitope markers might become part of the *O. volvulus* diagnostic toolbox.

**Electronic supplementary material:**

The online version of this article (10.1007/s00436-019-06345-3) contains supplementary material, which is available to authorized users.

## Introduction

Of the 20 infectious diseases listed on the World Health Organization (WHO) list of Neglected Tropical Diseases, eight of them are caused by a helminth infection (Holmes 2014; Hotez et al. 2008; WHO 2018). One of them, onchocerciasis (or river blindness) is caused by infection with the filarial nematode *Onchocerca volvulus*. The majority of infected people live in Africa, where at least 120 million are at risk (Borup et al. 2003; Enk 2006). Current treatment programs are based on mass drug administration (MDA) of the microfilaricidal agent ivermectin (Mectizan, Merck) as no approved macrofilaricide drugs or vaccines are available. However, contraindications in areas co-endemic for loiasis and an inability to break transmission in some foci ask for a change in strategy including vaccination in order to control or eliminate onchocerciasis (Hotez et al. 2015; Makepeace et al. 2015).

Traditionally, *Onchocerca* infection is diagnosed by detection of microfilariae (mf) in skin biopsy samples (skin snips) (Taylor et al. 1989). As this requires an invasive procedure, several efforts have been undertaken to identify novel biomarkers that offer a less-invasive, specific and sensitive marker for infection with *O. volvulus* (Vlaminck et al. 2015). Only one of these, the rapid-format test for the detection of IgG4 antibodies to the parasitic antigen Ov-16, has been further developed and is commercially available (Golden et al. 2016). This serological test is currently being used for elimination mapping of onchocerciasis (Dieye et al. 2017; Lont et al. 2017; Richards et al. 2018).

Recently, two peptide-based serology markers, OvMP-1 and OvMP-23, were introduced. Although they demonstrate promising characteristics in the limited sample set that was evaluated with OvMP-23 having high sensitivity (92.7%) and specificity (100%), further evaluation of their clinical utility is warranted before they can be used in the field (Lagatie et al. 2018). Other approaches that have shown promise are the use of specific metabolites in serum or urine (Denery et al. 2010; Globisch et al. 2013; Lagatie et al. 2016b; Shirey et al. 2018) and the detection of parasitic microRNAs in the blood of infected individuals (Lagatie et al. 2016a; Quintana et al. 2015; Tritten et al. 2014a; Tritten et al. 2014b).

In our previous work, the entire *O. volvulus* proteome was screened for the presence of linear epitopes, and 1110 immunoreactive peptides have been identified in this proteome, of which 249 were considered immunodominant, and as such can also be considered serodiagnostic candidates (Lagatie et al. 2017). In the study presented here, we investigated three of these immunoreactive peptides. Immune signals observed on the peptide microarray were confirmed by peptide ELISA and the epitope was mapped.

## Materials and methods

### Ethical statement

All samples used in this study were de-identified before being provided, and usage of these samples for research purposes was approved by an ethical committee or Institutional Review Board (IRB). For samples collected in Ghana, a study was approved by the Committee on Human Research, Publications and Ethics of the School of Medical Sciences of the Kwame Nkrumah University of Science and Technology, Kumasi, Ghana, and study subjects signed an informed consent form. For samples obtained from FR3, all were de-identified before they were provided to FR3, and usage of these samples for research purposes was approved by the Smith College Institutional Review Board (IRB). For samples collected in Belgium from healthy control donors, the Ethics Committee [“Commissie voor Medische Ethiek - ZiekenhuisNetwerk Antwerpen (ZNA) and the Ethics committee University Hospital Antwerp] approved the protocol, and informed consent, which was signed by all subjects. For samples obtained from Tissue Solutions Ltd. (Glasgow, Scotland), Discovery Life Sciences, Inc. (Los Osos, USA), Bioreclamation IVT (Baltimore, USA), Mayo Clinic (Jacksonville, USA), and Universitas Indonesia or University of Ghent, written informed consent was obtained from all individuals, and all samples were decoded and de-identified before they were provided for research purposes.

### Study samples

Plasma samples from *O. volvulus*-infected individuals were collected as part of a field study in Ghana. This study was undertaken in an onchocerciasis-endemic community located in Adansi South District along the Pra river basins in the Ashanti Region of Ghana. Physical examinations were performed to identify those subjects having palpable nodules. Skin snips (biopsies) were then taken in order to determine the microfilarial (mf) load in the skin (Debrah et al. 2015). Most subjects were participating in mass drug administration programs and had no or very low numbers (< 5 mf/mg skin) of microfilaria. Palpation is generally considered rather specific (estimated at 98% specific) but has somewhat lower sensitivity due to hidden nodules in deeper subcutaneous layers (Coffeng et al. 2013). No information was available on any other parasitic infections. A total of 93 nodule positive subjects that donated plasma samples were included (Table 1). Of those subjects, 63 were positive in the SD Bioline Ov16 IgG4 rapid test (i.e., 68%), which is slightly lower than the reported sensitivity of 80% which was determined using ELISA (Golden et al. 2016).

**Table 1:** Demographic information of study populations used for determination of diagnostic performance

Characteristic	Group										
	*O. volvulus infected*		Non-helminth infected						Helminth infected		
	*O. volvulus* Cameroon	*O. volvulus* Ghana	HC Southern Africa	HC Belgium	HIV	HCV	Dengue	Asthma	*Wb*	*Bm*	STH
Origin	Cameroon	Ghana	Southern Africa	Belgium	USA	USA	Vietnam	USA	Sri Lanka (8) Tahiti (2)	Indonesia (Central Sulawesi)	Ethiopia (Jimma)
No. of patients	8	93	10	49	25	25	25	25	10	20	24
Age, median (min-max)	49.5 (20–72)	43 (21–85)	21 (17–47)	40 (23–59)	n.a.	n.a.	26 (4–67)	46(17–91)	33 (13–48)	19 (10–45)	11.5 (5–18)
Gender, *n* (%)											
Male	5 (63)	50 (54)	7 (70)	22 (45)	n.a.	n.a.	10 (40)	11 (44)	6 (60)	10 (50)	13 (54)
Female	3 (37)	43 (46)	3 (30)	27 (55)	n.a.	n.a.	15 (60)	14 (56)	3 (30)	10 (50)	11 (46)
Unknown	0 (0)	0 (0)	0 (0)	0 (0)	0 (0)	0 (0)	0 (0)	0 (0)	1 (10)	0 (0)	0 (0)
Source^1^	FR3	KCCR	TS	Janssen	MC	MC	DLS	BR	FR3	UI	UG
Ov16 IgG4 positive, *n*	8	63	0	0	0	0	0	1	0	0	0

^*1*^*FR3*, Filariasis Research Reagent Resource Center; *KCCR*, Kumasi Centre for Collaborative Research; *TS*, tissue solutions; *MC*, Mayo Clinic; *DLS*: Discovery Life Sciences; *BR*, bioreclamation; *UI*, Universitas Indonesia; *UG*, University of Ghent

*n.a.:* not available; *Wb*, *Wuchereria bancrofti*; *Bm*, *Brugia malayi*; *STH*, soil-transmitted helminths

Additionally, a second set of eight serum samples from *O. volvulus*-infected individuals, collected in Cameroon by Dr. Nutman, was obtained through the Filariasis Research Reagent Resource Center (FR3), Division of Microbiology and Infectious Diseases, NIAID, NIH. Information on *O. volvulus* infection (number of microfilaria/mg skin and number of palpable nodules) was provided by FR3, along with demographic information (Table 1). All infected individuals had at least two palpable nodules and 25 mf/mg skin (microfilaridermia) as determined by skin snip and were positive in the Ov16 IgG4 rapid test.

For the non-helminth-infected control samples, demographic information is also provided in Table 1. A first healthy control sample set was composed of nine serum samples from individuals from Southern Africa, and was provided by Tissue Solutions Ltd. (Glasgow, Scotland). A second healthy control sample set was composed of 49 plasma samples from healthy individuals from Belgium. (Lagatie et al. 2014a; Lagatie et al. 2014b; Lagatie et al. 2014c; Stuyver et al. 2013; Van Loy et al. 2013). Furthermore, different sets of plasma samples were obtained from Discovery Life Sciences, Inc. (Los Osos, USA), Bioreclamation IVT (Baltimore, USA), FR3, or Mayo Clinic (Jacksonville, USA).

For the cross-reactivity panels (non-*Onchocerca* helminth-infected individuals), a set of ten samples from *Wuchereria bancrofti*-infected individuals was obtained through the Filariasis Research Reagent Resource Center (FR3), Division of Microbiology and Infectious Diseases, NIAID, and NIH. Samples were collected in Tahiti by Dr. Perolat (Institut Territorial de Recherches Médicales Louis Malardé, Tahiti, Polynésie Française) or Sri Lanka by an unknown collector, and information on *W. bancrofti* infection (number of microfilaria/mL) was provided by FR3, along with demographic information (Table 1). One set of 20 samples from individuals with *Brugia malayi* infection, collected in Central Sulawesi, Indonesia was kindly provided by Prof. Yazdanbakhsh (Leiden University Medical Center, Leiden, The Netherlands). A last sample set from 24 individuals infected with soil-transmitted helminths (STH) and/or *Schistosoma mansoni*, collected in Jimma, Ethiopia, was kindly provided by Prof. Levecke (Ghent University, Merelbeke, Belgium) (Table 1). STH infections included *Ascaris lumbricoides*, *Trichuris trichiura*, and hookworm (*Necator americanus*), either as single or mixed infected.

### Total IgG peptide ELISA

Biotinylated synthetic peptides were synthesized by standard procedures and purchased from PEPperPRINT GmbH (Heidelberg, Germany). Peptides that only contain the minimal epitopes flanked at both termini by a small linker peptide (Val-Ser-Val) were designated OvNMP for *O. volvulus* non-motif peptide (Table 3). For determination of peptide-specific serum antibody levels, peptide ELISA was developed and set up as described previously (Lagatie et al. 2017). For visualization purposes, all data points that had background-corrected absorbance below 0.01 were replaced by 0.005 to enable proper plotting on a logarithmic scale.

### Epitope mapping

Permutation scans were carried out by PEPperPRINT GmbH (PEPperCHIP® Platform Technology, Heidelberg, Germany). In a permutation scan, the effect on binding of replacing each of the amino acid residues by all amino-acids is analyzed, which requires the synthesis of 20*x spot peptides per starting peptide (20 amino-acids at x positions, with x the length of the peptide). Seven arrays were prepared, each containing the permutation analysis of the WT peptide and of 19 variants. Three thousand three hundred twenty different peptides were printed at least in duplicate and were framed by additional Influenza Hemagglutinin (HA) (YPYDVPDYAG) and polio (KEVPALTAVETGAT) control peptides (186 spots for each control). Each of the peptide arrays was incubated with 500-fold diluted serum sample and stained with Goat anti-human IgG (Fc) DyLight680 (1:5000) and goat anti-human IgM (μ chain) DyLight800 (1:5000). Arrays were scanned with LI-COR Odyssey Imaging System, and fluorescence signals were used to calculate relative intensity compared to the native peptide. Consensus epitopes were derived from these analyses whereby the consensus epitope is defined as the region in the peptide for which a strong and consistent reduction in the signal is observed when a single amino acid is replaced by any other or a non-related amino acid.

### Statistical analysis

For the minimal epitope peptides investigated in this study, ROC analysis was performed. Several sample sets from non-helminth-infected individuals were used as control group. These include healthy controls from both Belgium and South Africa, HIV-infected or HCV-infected individuals from USA, dengue-infected individuals from Vietnam, and asthma patients from the USA. Two groups of *O. volvulus*-infected individuals were used as positive group: one from Cameroon and one from Ghana. ROC analysis was performed using all “non-helminth controls” vs. “all *Ov*-positive samples” and cutoffs were determined as the point with maximal specificity, at the expense of a decrease of sensitivity. In the case of OvNMP-48, the cutoff was defined as the point with maximal Youden’s index ((sensitivity + specificity)-1). Based on these cutoffs, sensitivity and specificity of each peptide ELISA were determined, as well as cross-reactivity with other helminths in samples with *Brugia*, *Wuchereria*, or STH infections. All analyses were performed using GraphPad Prism 7.

## Results

### Confirmation of peptide microarray using peptide ELISA

From the list of 1110 immunoreactive peptides, three were selected for further evaluation (results not shown) and peptide ELISA was developed. Immune reactivity against these peptides was determined in a random set of nodule-positive individuals (*n* = 50) and healthy controls (*n* = 38). Of importance, for both onchocerciasis patients and healthy controls, samples were different than those used in the peptide arrays (Lagatie et al. 2017). To be able to compare the performance of these peptides, an arbitrary cutoff was defined at 0.1, and sensitivity and specificity were calculated for all peptides (Table 2). All three peptides were confirmed to be recognized by the antibodies in at least a subset of nodule-positive individuals.

**Table 2:** Performance characteristics of the selected peptides in ELISA

Peptide ID	Peptide sequence	Sensitivity (%) (*n* = 50)	Specificity (%) (*n* = 38)	Median signal nodule positives (+IQR)
OVOC10067;2131	TVEGGDNNGANFE	24.0	100	0.02 (0.00–0.08)
OVOC1858;283	INRDANLNANSNPND	35.0	100	0.06 (0.02–0.15)
OVOC2814;121	SDWDSEKDGKKKD	44.0	100	0.08 (0.02–0.17)

*IQR* interquartile range

### Determination of the minimal epitope

To determine the key amino acids responsible for immune recognition, epitope mapping of these three peptides was performed (Table 3). Peptide microarrays covering full substitution scans of all four peptides, in which all amino acid positions were substituted by the 19 other amino acids, were synthesized and analyzed using seven samples from nodule-positive individuals. For every single peptide and every sample, an amino acid plot was calculated (Fig. S1) and for each peptide, the consensus epitope is given in Table 3. Unfortunately, not all samples had sufficiently high reactivity for all peptides, resulting in the empty plots in Fig. S1. Although some minor differences exist between the different samples, the minimal epitopes of the peptides could be determined. For all three peptides, the epitopes do not share any similarity with previously described epitopes (Lagatie et al. 2017; Lagatie et al. 2018).

**Table 3:** Epitope mapping and sequence of peptides based on the minimal epitopes

Epitope mapping			Minimal epitope peptides	
Peptide ID	Peptide sequence	Epitope	Peptide name	Sequence
OVOC10067;2131	TVEGGDNNGANFE	DNNGANFE	OvNMP-14	VSV- DNNGANFE-VSV-Biotin
OVOC1858;283	INRDANLNANSNPND	NLNANSNPN	OvNMP-16	VSV- NLNANSNPN-VSV-Biotin
OVOC2814;121	SDWDSEKDGKKKD	EKDGKK	OvNMP-18	VSV- EKDGKK-VSV-Biotin

### Characterization of peptides with minimal epitopes

Peptides were synthesized that only contain the minimal epitopes flanked at both termini by a small linker peptide (Val-Ser-Val). To determine the analytical performance of these peptides (sensitivity and specificity), reactivity of these peptides was assessed in sample sets from *O. volvulus*-infected individuals and different sets of non-helminth-infected individuals (Table 4). As a reference, also OvMP-1 and OvMP-23 are included in this table (Lagatie et al. 2018). For all three peptides, the specificity/sensitivity profile in ROC analysis allowed to define a cutoff that corresponded to 100% specificity, which makes them attractive markers for further evaluation.

**Table 4:** Performance characteristics of *O. volvulus* minimal epitope containing peptides

	Cutoff^1^	Sensitivity (%) (*n* = 101)	Specificity (%) (*n* = 158)	Cross-reactivity (%) (*n* = 54)	Median signal nodule positives (+IQR)
OvMP-1	0.045	100	98.7	72.0	3.39 (2.67–3.62)
OvMP-23	0.11	92.7	100	6.0	0.64 (0.30–1.26)
OvNMP-14	0.105*	36.9	100	5.6	0.06 (0.02–0.20)
OvNMP-16	0.085*	46.5	100	5.6	0.07 (0.02–0.15)
OvNMP-18	0.115*	41.2	100	9.3	0.08 (0.01–0.27)
OvNMP-48	0.118	76.0	97.5	11.1	0.29 (0.12–0.73)

^1^Background-corrected absorbance

*Cutoff corresponding to 100% specificity

*IQR* interquartile range

**Fig. 1:**
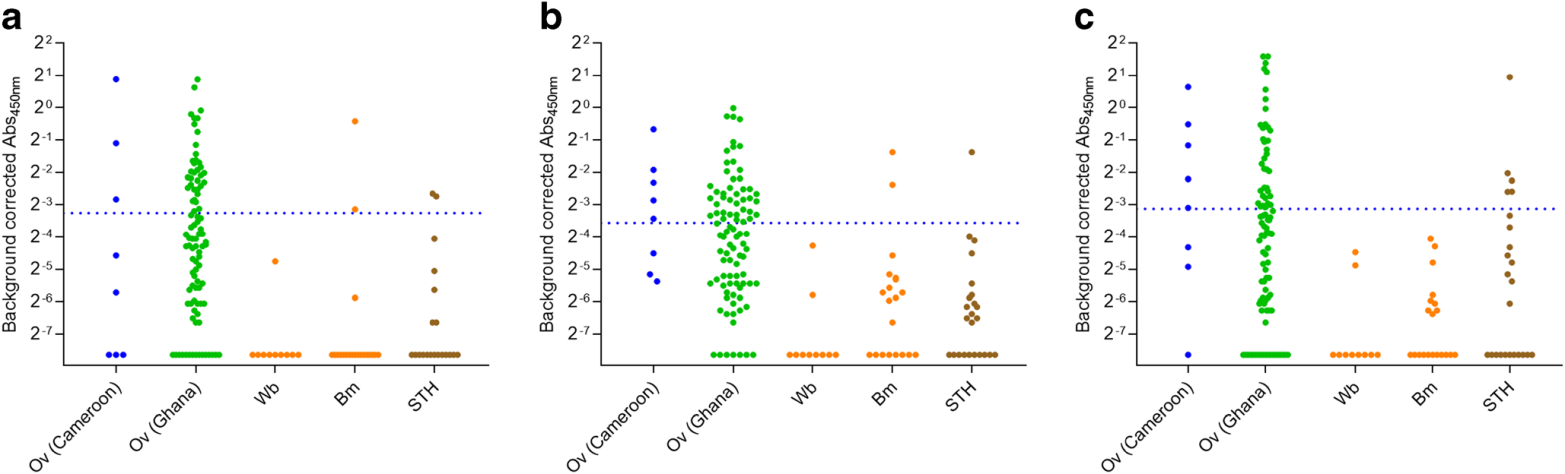
Assessment of cross-reactivity against minimal epitope peptides. Immunoreactivity against OvNMP-14 (**a**), OvNMP-16 (**b**), and OvNMP-18 (**c**) was determined in helminth-infected individuals. Dotted lines indicate the cutoff determined by ROC analysis. All samples used in this graph are described in Table 1

As cross-reactivity with other helminth infections is an important issue in serological assays, we also investigated the reactivity of these peptides in sample sets from non-*Onchocerca* helminth-infected individuals (Table 4 and Fig. 1). The data demonstrate that, although all three peptides have 100% specificity, they display slight cross-reactivity with other parasite infections (5.6%, 5.6%, and 9.3%, respectively). Also, their sensitivity is too low to be useful as a standalone diagnostic marker (36.9%, 46.5%, and 41.2%, respectively).

### Combination of different epitopes in one peptide

**Fig. 2:**
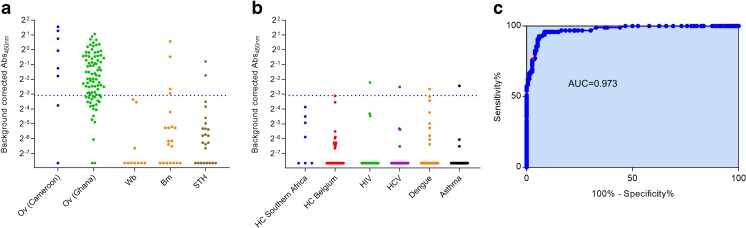
Performance of a peptide with three epitopes in peptide ELISA. Immunoreactivity against OvNMP-48 was determined in helminth-infected individuals (**a**) and non-helminth-infected individuals (**b**). Dotted lines indicate the cutoff determined by ROC analysis. **c** ROC analysis of onchocerciasis versus non-helminth infected individuals, with indication of the area under curve (AUC). All samples used in this graph are described in Table 1

As we demonstrated before, it is possible to combine different epitopes in one peptide to further optimize the performance of the assay (Lagatie et al. 2018). We therefore tested a peptide consisting of the three epitopes from OvNMP-14, OvNMP-16, and OvNMP-18, separated by a small linker (VSV-DNNGANFE-VSV-NLNANSNPN-VSV-EKDGKK-VSV, called OvNMP-48). Like the single epitope peptides, a specific cutoff was defined based on ROC analysis, resulting in a 97.4% specificity, 76.0% sensitivity, and a cross-reactivity of 11.1% (Table 4. Cross-reactivity was not attributed to a specific helminth infection as 4 out of 20 *B. malayi* and 2 out of 24 STH-infected individuals were found to be positive and none of the ten investigated samples from *W. bancrofti*-infected individuals was found to be positive. Some of the non-helminth-infected individuals also had an outlying positive response (Fig. 2).

## Discussion

In this work, we have evaluated three immunoreactive *O. volvulus* peptides. We confirmed the peptide microarray data using peptide ELISA; epitope mapping was performed, and peptides were constructed that contained only the minimal epitope, flanked by a linker. Investigation of the performance of these minimal epitope peptides demonstrated that all three of them have a specificity of 100%, low cross-reactivity (< 10%), but rather low sensitivity (< 50%). Assessment of the performance of a combination of these three epitopes in one peptide, called OvNMP-48 showed that sensitivity could be further increased to 76.0%, but with a small effect on specificity. It must be emphasized that for the control population, no information is available on any helminth infection and that therefore it cannot be excluded that some of these individuals had been exposed to helminths (and even *O. volvulus*), causing some reactivity against this peptide. The results demonstrate that combination of different linear epitopes, even if they have rather poor sensitivity, can result in a test that has a sufficiently good sensitivity/specificity profile to encourage further exploration and optimization of the epitopes and ultimately evaluation of its clinical utility. Besides the obvious increase in sensitivity, this result also demonstrates the possible drawback of combining multiple epitopes in one peptide as cross-reactivity of the different epitopes is now also reduced to a single result without information on which and how many epitopes are recognized by the antibodies in the sample. The use of multiple individual antigens in a classifier (immunosignature) might be better suited to overcome this issue as has been demonstrated before for Chagas disease (Zrein et al. 2018). An alternative strategy whereby multiple peptides are used in a mixture as capturing antigen was also shown to be a promising strategy to optimize sensitivity and specificity (Dubois et al. 2012). More work will be needed to investigate the performance of these linear epitopes as biomarker, either individually, as part of an immunosignature, or as combination in one peptide. Especially the assessment of cross-reactivity with other helminth species that are co-endemic with *O. volvulus*, such as *Loa loa*, *Mansonella,* and *Strongyloides*, will be essential.

## Electronic supplementary material

Figure S1 Epitope mapping details presented by amino acid plots. The amino acid plots were calculated by dividing the spot intensity of a given peptide (e.g. ^1^YPYDVQDYA^9^) by the spot intensity of the native epitope (^1^YPYDVPDYA^9^). The position of an amino acid at a given position, thus, reflected the intensity ratio compared to the amino acid of the native epitope at the same position. In case the native epitope had too low signal in a particular sample, then no amino acid plot could be generated. (DOCX 1010 kb)

## Abbreviations

*O. volvulus*: *Onchocerca volvulus*
mf: microfilaria
WHO: World Health Organization
MDA: Mass Drug Administration
ELISA: enzyme-linked immunosorbent assay
IRB: Institutional Review board
ROC: Receiver Operating Characteristic
