# Supplementary figures and images for: Performance evaluation of 3 serodiagnostic peptide epitopes and the derived multi-epitope peptide OvNMP-48 for detection of Onchocerca volvulus infection
Source: Parasitol Res. 2019 May 14;118(7):2263–70. doi: 10.1007/s00436-019-06345-3 (PMC6611744; doi:10.1007/s00436-019-06345-3)

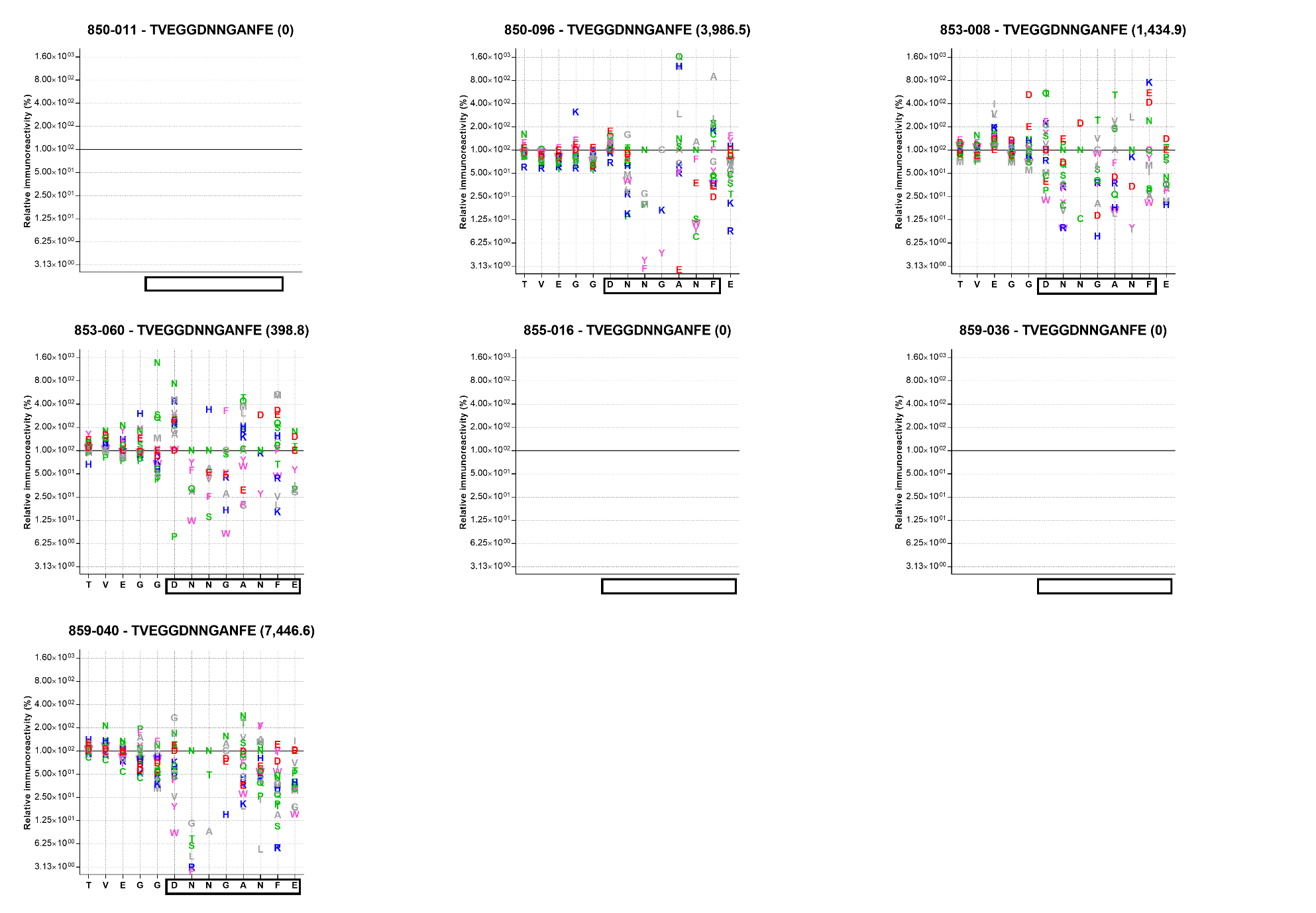

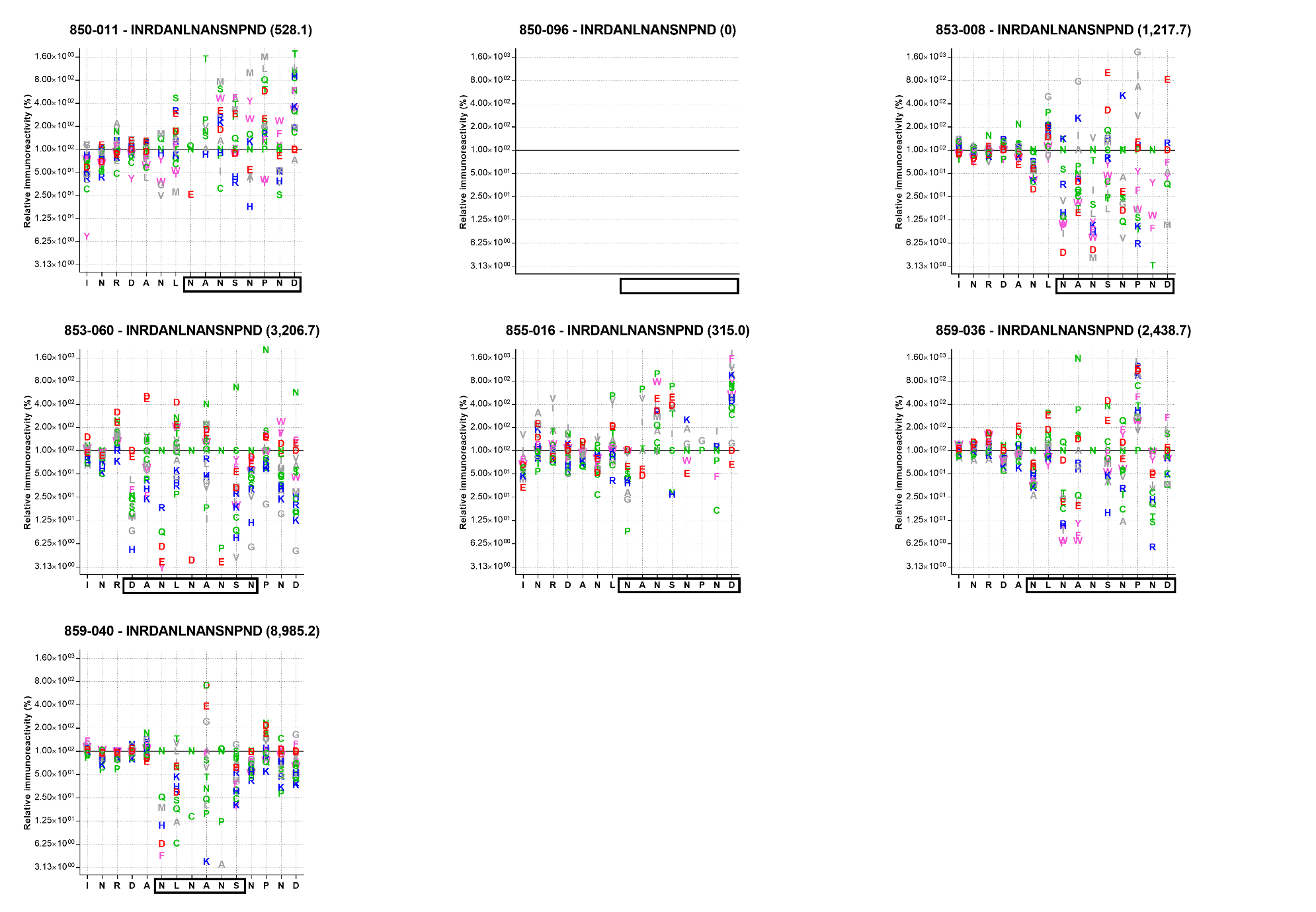

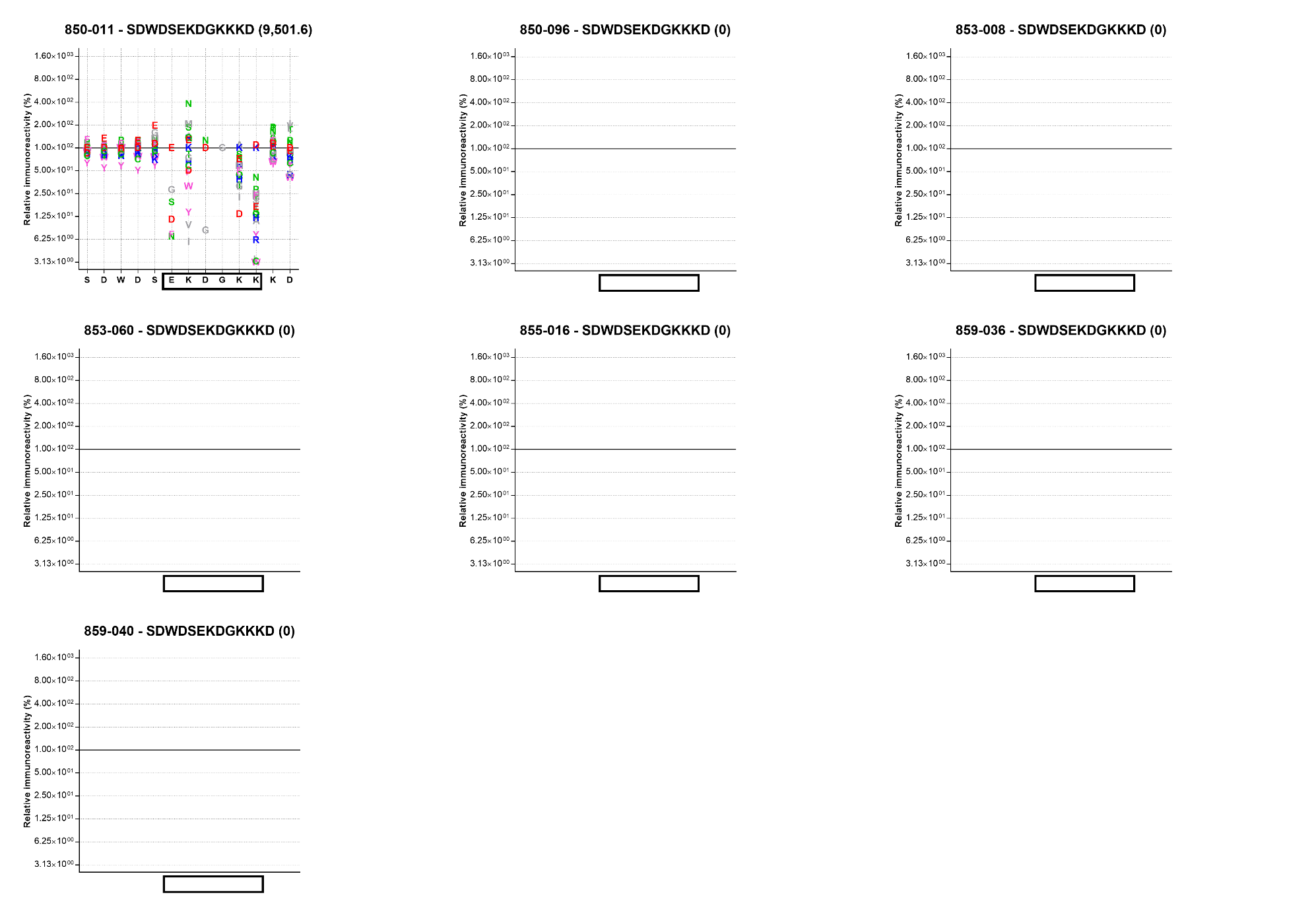

Supplement: Supplementary file 1 — Epitope mapping details presented by amino acid plots. The amino acid plots were calculated by dividing the spot intensity of a given peptide (e.g. 1YPYDVQDYA9) by the spot intensity of the native epitope (1YPYDVPDYA9). The position of an amino acid at a given position, thus, reflected the intensity ratio compared to the amino acid of the native epitope at the same position. In case the native epitope had too low signal in a particular sample, then no amino acid plot could be generated. (DOCX 1010 kb) [file 436_2019_6345_MOESM1_ESM.docx]
